# Co-design and evaluation of a regional and rural translation bronchiolitis platform: study protocol

**DOI:** 10.1038/s41390-025-04503-1

**Published:** 2025-11-03

**Authors:** Louise Shaw, Catherine Wilson, Meredith Borland, Elizabeth Cotterell, John Craven, Janet Curran, Stuart Dalziel, Tahnee Dunlop, Donna Franklin, Libby Haskell, Anna Lithgow, Sandy Middleton, Sharon O’Brien, Annelise Staples, Viet Tran, Franz E. Babl, Emma Tavender

**Affiliations:** 1 Emergency Research, Murdoch Children’s Research Institute, Melbourne, VIC Australia; 2 Centre for Digital Transformation of Health, University of Melbourne, Melbourne, VIC Australia; 3 Emergency Department, Perth Children’s Hospital, Nedlands, WA Australia; 4 Divisions of Emergency Medicine and Paediatrics, School of Medicine, University of Western Australia, Crawley, WA Australia; 5 Department of Paediatrics, Armidale Rural Referral Hospital, Armidale, NSW Australia; 6 School of Rural Medicine, University of New England, Tablelands Clinical School, Armidale, NSW Australia; 7 Emergency Department, Mount Barker District Soldiers Memorial Hospital, Mount Barker, SA Australia; 8 College of Medicine and Public Health, Flinders University, Bedford Park, SA Australia; 9 Faculty of Health, Charles Darwin University, Darwin, NT Australia; 10 Faculty of Health and Medical Sciences, The University of Adelaide, Adelaide, SA Australia; 11 School of Nursing, Dalhousie University, Halifax, NS Canada; 12 Departments of Surgery and Paediatrics, The University of Auckland, Auckland Central, New Zealand; 13 Emergency Medicine Research, Children’s Emergency Department, Starship Children’s Hospital, Auckland, New Zealand; 14 Centre for Rural Emergency Medicine, Deakin University, Warrnambool, VIC Australia; 15 Children’s Emergency and Critical Care Research, Gold Coast University Hospital, Southport, QLD Australia; 16 Menzies Health Institute Queensland, Griffith University, Gold Coast, QLD Australia; 17 College of Medicine and Dentistry, James Cook University, Townsville, QLD Australia; 18 Department of Paediatrics: Child and Youth Health, University of Auckland, Auckland, New Zealand; 19 Paediatric Department, The Royal Darwin Hospital, Tiwi, NT Australia; 20 Nursing Research Institute, St Vincent’s Health Network, Darlinghurst, NSW Australia; 21 School of Nursing, Midwifery and Paramedicine, Australian Catholic University, Fitzroy, VIC Australia; 22 Bendigo Health Emergency Department, Bendigo, VIC Australia; 23 Tasmanian School of Medicine, University of Tasmania, Hobart, TAS Australia; 24 Royal Hobart Hospital, Tasmanian Health Service, Hobart, TAS Australia; 25 Tasmanian Emergency Medicine Research Institute, University of Tasmania, Hobart, TAS Australia; 26 Emergency Department, The Royal Children’s Hospital Melbourne, Melbourne, VIC Australia; 27 Departments of Paediatrics and Critical Care, University of Melbourne, Melbourne, VIC Australia; 28 Faculty of Health Sciences, Australian Catholic University, East Melbourne, VIC Australia

## Abstract

**Background:**

Bronchiolitis is the leading cause of hospitalisation in infants under one year. While evidence-based guidelines exist, including the updated Australasian Bronchiolitis Guidelines (2025), variations in care persist. Improving care in regional and rural hospitals is challenging due to limited access to paediatric expertise and evidence-based resources, contributing to continued use of low-value interventions.

**Methods:**

This mixed methods study will use a human-centred design approach to co-design and evaluate the usability of the Regional and Rural Translation Bronchiolitis (RART-Bronch) platform, an interactive online tool targeted at non-metropolitan settings. The platform will feature bronchiolitis educational resources, implementation support, a benchmarking and feedback tool, and family education materials. Participants will be regional and rural clinicians and parents of infants hospitalised with bronchiolitis in these settings. Data will be collected through co-design meetings, usability surveys, semi-structured interviews, and think aloud methods. Engagement with the co-design process will also be evaluated.

**Results:**

We will develop a user-friendly, evidence-based platform specific to regional and rural contexts that supports bronchiolitis guideline adherence and enhances clinical decision-making.

**Conclusion:**

Effectiveness will be evaluated in a future cluster randomised controlled trial and may inform future implementation strategies for improving care, quality and equity across regional and rural healthcare.

**Impact:**

- This study aims to reduce variation in bronchiolitis care by co-designing an interactive online platform, RART-Bronch, with clinicians and parents from regional and rural settings.
- The platform aims to support sustainable reductions in low-value care and improve outcomes for infants with bronchiolitis.
- It will deliver evidence-based guidance to clinicians working in regional and rural hospitals with limited paediatric expertise.
- Findings will inform future implementation of tailored education, audit and feedback strategies in acute care.
- This work aims to enhance paediatric care quality in under-resourced settings and contribute to scalable, equitable improvements in bronchiolitis management across Australia.

## Introduction

Bronchiolitis is the leading cause of hospitalisation in infants under one year of age, accounting for significant morbidity and healthcare resource utilisation worldwide.^1^ It is a viral infection that affects the small airways causing respiratory distress and feeding difficulties, often requiring utilisation of hospital services for diagnosis, investigation and management.^1–9^ Management primarily involves supportive care around adequate oxygenation and hydration, as bronchiolitis is generally a predictable and self-limiting illness.^4^ However, between 10 and 20% of infants require escalation of care, including transfer to tertiary hospitals for intensive respiratory support.^1,10,11^ These inter-hospital transfers can be especially challenging in regional and rural settings, where on-site paediatric expertise and high-acuity services are often limited. The decision to transfer can be complex for clinicians managing care with limited resources, and distressing for families, who may face significant emotional, financial, and logistical burdens during transfer. This study responds to the pressing need for context-specific strategies that can improve the management of children with bronchiolitis in these non-metropolitan settings.

Despite the availability of robust, evidence-based guidelines for bronchiolitis management,^1–9^ significant variation in care persists. Up to 40% of infants hospitalised with bronchiolitis receive care known to be ineffectual, such as unnecessary use of antibiotics, salbutamol, corticosteroids, adrenaline, and chest X-rays.^1,12^ While often well-intended, these low-value interventions and diagnostic tests contribute to increased healthcare costs, unnecessary hospital stays, and potential patient harm.^13–15^ Another area that merits targeted improvement efforts is the increasing use of high-flow nasal cannula (HFNC) therapy. Although HFNC has been demonstrated to rescue hypoxic infants failing standard oxygen therapy in 60 per cent of cases,^16^ its use has extended beyond the evidence-based indications. Studies from both Australia and the United States have reported a marked increase in the use of HFNC therapy in the management of bronchiolitis.^17,18^ This increase has occurred despite multiple randomised controlled trials showing that early or routine use of HFNC in infants with mild to moderate bronchiolitis does not reduce the need for intubation or shorten hospital length of stay.^16,19,20^ The growing reliance on HFNC highlights a disconnect between clinical practice and the current evidence base, raising concerns about the potential for overuse and the delivery of low-value care. These trends illustrate the need for implementation strategies that are context-specific and responsive to the realities of rural and regional healthcare delivery.

Successful efforts to reduce variations in care have leveraged implementation science to systematically improve and sustain evidence-based practices. Evidence suggests that targeted interventions, those specifically designed to address identified barriers and leverage enablers within a given context, are most effective.^21–23^ One such targeted strategy is audit and feedback—a continuous process of benchmarking clinical practice against established standards (audit) and providing the resulting data to health professionals (feedback). Audit and feedback mechanisms have been shown to drive practice changes in acute care settings,^24^ including in the management of bronchiolitis.^21,25^ A cluster randomised controlled trial (cRCT) involving 26 Australasian hospitals (seven tertiary paediatric hospitals and 19 large mixed hospitals), demonstrated that targeted implementation interventions reduced the use of ineffective and potentially harmful therapies and management strategies, decreased unnecessary interventions, and improved bronchiolitis care.^22^ Subsequent feasibility testing of these implementation interventions in four different grades of hospitals in Western Australian replicated these findings, confirming that they could be adapted outside of a controlled trial setting.^21^ However, regional and rural-based clinicians may need additional support to effectively implement evidence-based bronchiolitis management, particularly in settings with limited resources and workforce constraints.

## Scaling implementation strategies in regional and rural settings

While evidence-based implementation interventions have been successfully applied in tertiary and metropolitan hospitals, translating and scaling these strategies to regional and rural settings remains a significant challenge. Healthcare professionals in non-tertiary hospitals often lack access to targeted educational resources, and peer benchmarking and feedback systems to support sustainable practice change.^26,27^ Implementation platforms and toolkits offer a scalable, cost-effective approach to addressing these challenges,^28^ yet there is limited research on their effectiveness in acute care settings outside of large metropolitan centres.

## The RART-Bronch platform

**Fig. 1:**
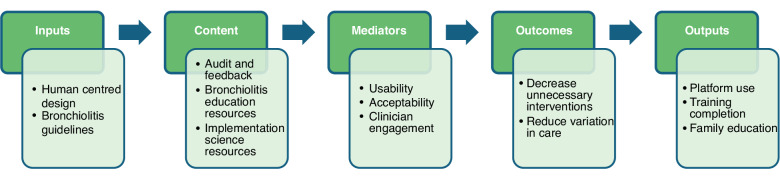
Schematic overview of the RART-Bronch interactive online platform, showing the inputs, content, mediators, outcomes and outputs.

To address these challenges, this study will co-design and evaluate the usability of a RART-Bronch platform, an interactive online tool targeted to the needs of regional and rural hospitals. Figure 1 represents a schematic overview of the platform’s inputs, components, mediators and outputs. Co-designed with clinicians, parents, and caregivers from non-metropolitan settings, the platform will build on previous bronchiolitis improvement efforts and incorporate co-design principles and evidence from implementation science, including audit and feedback mechanisms. The RART-Bronch platform aims to bridge the gap between clinical evidence and practice, ensuring equitable access to high-quality care for infants with bronchiolitis across regional and rural settings. The study will also contribute new insights on how to effectively co-design online materials for regional and rural health settings, to inform future improvement efforts.

## Study aims and objectives

The aim of this study is to co-design and evaluate the usability testing of a RART-Bronch platform to improve the management of infants with bronchiolitis in regional and rural hospitals. The platform is designed primarily for clinicians and will provide contextually targeted implementation support, educational materials, and real-time audit and benchmarking tools to facilitate evidence-based bronchiolitis management in regional and rural hospitals. Co-designed family-facing educational materials will also be included. These are intended to be used by clinicians during interactions with parents to enhance clinician-parent communication, strengthen discharge communication processes, and support parents in caring for their infants at home, helping to reduce unnecessary hospital visits and alleviate anxiety related to bronchiolitis management.

Upon completion, the RART-Bronch platform will be implemented and evaluated in a cluster randomised controlled trial (cRCT) across 30 regional and rural hospitals, to evaluate its impact on clinical practice and patient outcomes. A separate protocol will be developed for the cRCT.

## Methods

### Study design

This study will use a mixed-methods, human-centred design approach to co-design and evaluate the usability of the RART-Bronch platform for improving bronchiolitis management in regional and rural hospitals. The RART-Bronch platform is being developed as a stand-alone web-based microsite, hosted within the research organisation’s website. Co-designed with clinicians, it will provide bronchiolitis educational resources, implementation science training materials, and an audit and feedback function that enables hospitals to enter de-identified data and receive automated benchmarking reports against evidence-based indicators. Parent-facing discharge materials will be co-designed with parents and made available on the platform for clinicians to use when communicating with families.

**Fig. 2:**
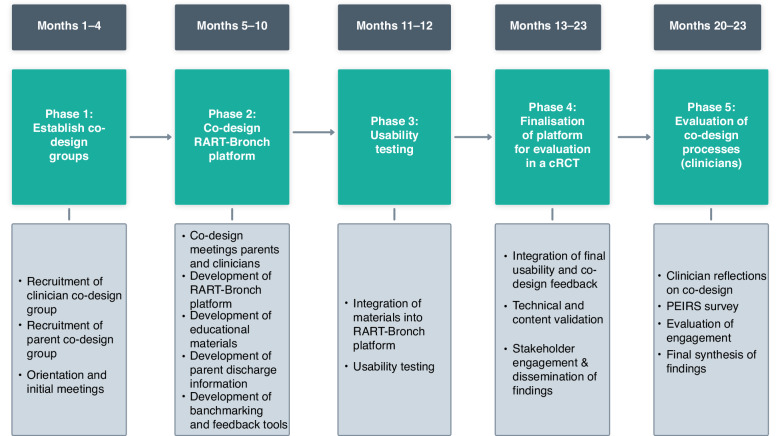
RART-Bronch study phases and timeline.

The study is structured into five phases, each focusing on a distinct stage of platform development and evaluation. These phases integrate qualitative and quantitative data collection to ensure iterative refinement and optimisation of the platform before broader implementation. The study phases and timeline are outlined in Fig. 2.

The research process is informed by implementation science, human factors design principles, and evidence-based audit and feedback strategies. The human-centred co-design methodology will engage clinicians and parents, ensuring that the platform’s features align with end-user needs and can be feasibly integrated into clinical workflows. This study will also assess the usability and acceptability of the platform through structured usability testing, leading to refinements that optimise functionality and sustainability. The study’s final phase will involve an evaluation of the co-design process, generating insights into best practices for co-designing online education and implementation support materials for regional and rural acute care settings.

Ethical approval for the study was obtained from the Human Research Ethics Committee at The Royal Children’s Hospital, Melbourne.^29^

### Participants and recruitment

The study will involve three participant groups. All participants will provide informed consent to participate. Recruitment for the co-design groups is expected to take place in June and July 2025. Usability testing clinicians will be recruited prior to commencement of usability testing. Table 1 outlines the participant groups involved in the co-design and usability testing of the RART-Bronch platform. It details the roles, eligibility criteria, and contributions of clinicians and parents to ensure the platform meets both clinical and family needs.

**Table 1:** Participants, roles and eligibility criteria

Participant group	Participants	Role	Eligibility criteria
Co-design clinicians	5 nurses and 5 doctors from different Australian states and territories who work in regional or rural hospital Emergency Departments or short stay inpatient wards, and care for infants with bronchiolitis.	Co-design platform content including educational materials and audit/feedback components and participate in usability testing	• Junior or senior medical or nursing staff working in a regional or rural ED or short stay inpatient ward. • Experience in managing infants with bronchiolitis. • Able to participate in online co-design and usability meetings. • Provide written informed consent via an online platform.
Usability testing clinicians	An additional 5 nurses and 5 doctors from different Australian states and territories who work in regional or rural hospital Emergency Departments or short stay inpatient wards, and care for infants with bronchiolitis.	Participate in usability testing	• Junior or senior medical or nursing staff working in a regional or rural ED or short stay inpatient ward. • Experience in managing infants with bronchiolitis. • Able to participate in online usability testing meeting. • Provide written informed consent via an online platform.
Co-design parents	7–14 parents from regional or rural areas who visited the ED with a child (<12 months of age at the time of presentation) with bronchiolitis in the last 18 months.	Co-design parent-facing content, such as discharge information and other educational materials.	• Live in a regional or rural area. • Have visited a regional or rural ED with their infant (< 12 months of age at the time of presentation) with bronchiolitis in the past 18 months. • Able to participate in online co-design meetings. • Provide written informed consent via an online platform
First Nations community consultation	Indigenous liaison officers will facilitate consultation with parents and carers through existing regional and rural community groups (e.g., ‘mums and bubs’ gatherings)	Provide culturally appropriate feedback on parent-facing discharge and educational materials.	• Parents and carers participating in community gatherings by Indigenous liaison officers, • Able to participate in face-to-face meetings within community settings • Able to provide feedback to refine materials for cultural relevance and accessibility,

#### Clinician recruitment

Recruitment of clinicians will be performed using convenience sampling by inviting clinicians who work in a regional or rural Emergency Department that manages infants with bronchiolitis. Clinicians will be recruited through professional networks and hospital contacts via the Paediatric Research in Emergency Departments International Collaborative (PREDICT) network.^30,31^ Potential participants will be contacted through their affiliated hospitals and invited via email distribution lists. If the initial recruitment target is not met within four weeks, the study will be advertised more broadly to eligible clinicians in regional and rural hospitals. To prevent contamination of the subsequent cRCT, clinicians from hospitals participating in the trial will not be eligible for the platform development. Clinicians agreeing to participate in the platform development will have the option of taking part in the co-design process and usability testing phases, or the usability testing phase only.

#### Parent recruitment

Convenience sampling will be used for the parent co-design group aiming to attract a diverse group in terms of sex, ethnicity, health literacy, and geographic distribution across different states and territories. Parents will be eligible if they live in a regional or rural community and have presented to an emergency department with their infant (aged under 12 months) for bronchiolitis treatment in the preceding 18 months. Parents will be recruited through parent support networks, hospital clinician approach, and consumer advocacy groups. A recruitment poster will be distributed through relevant organisations.

Baseline demographic data will be collected following participant enrolment via a brief questionnaire on REDCap, after completing consent. For parents, this will include the Brief Health Literacy Screening (BRIEF) tool and first language. To ensure First Nations input, we are liaising with Indigenous liaison officers in regional and rural areas to guide culturally appropriate consultation and engagement. This will involve working with established community groups to guide consultations with First Nations parents, supporting community-based feedback processes. These strategies are intended to establish engagement from the outset of the project.

Interested parent and clinician participants will be encouraged to contact the study coordinator for further information and will be provided with an information summary outlining the study. Participants will be excluded if they are unable to complete meetings in English, lack internet access for participation, or do not meet the inclusion criteria. Once eligibility is confirmed, interested participants will receive an email containing the Plain Language Statement and a link to the online consent form via Research Electronic Data Capture (REDCap), a secure electronic data capture platform hosted at [blinded for review].^32,33^

### Study phases: specific methods and data collection

The study will be conducted over five sequential phases (Fig. 2), guided by participatory co-design principles, to design the RART-Bronch platform and evaluate its usability in collaboration with clinicians and parents; each phase is outlined in detail below.

### Phase 1: Establishing co-design groups (Months 1–4)

In this phase, the two co-design participant groups (clinicians and parents), will be established (Table 1). Participants will complete an initial demographic questionnaire via REDCap. For clinicians, data collected will capture role and years of experience. For parents, data will cover assessment of health literacy, first language, and whether they are of Aboriginal or Torres Strait Islander origin.

### Phase 2: Co-designing the RART-Bronch platform (Months 5–10)

Phase 2 of the project will focus on the co-design of the RART-Bronch platform with users, over a 5-month period. Both participant groups will engage in structured online discussions via Microsoft Teams, facilitated by the research team. A human-centred design approach will be employed, informed by implementation science, human factors design principles, and existing literature on effective audit and feedback mechanisms. A multi-method approach will be used to investigate users’ needs and preferences, ensuring the platform is designed for integration and long-term sustainability in clinical practice.

Co-design meetings will focus on collaboratively developing and refining the RART-Bronch platform, developing implementation and educational content, and parent-facing materials through iterative discussion, feedback, and shared decision-making between clinicians and parents. Table 2 provides an overview of the meetings that will be conducted during the co-design and evaluation phases of the RART-Bronch platform development, detailing the focus of each session, participant groups, format, duration, and content discussed.

**Table 2:** Schedule for qualitative meetings

Phase of study	Platform content	Meeting focus	Participants	Individual (I) or group (G)	Meeting number	Meeting leader	Type of meeting (face to face vs video)	Length of meeting	Meeting content overview
**Phase 2:** Co-design of the RART-Bronch platform with users	1. Implementation support and Educational Content	Initial content development	Co-design clinicians	G	1	PI	Microsoft Teams	60 mins	Review content, provide feedback on format, length, and usability
	1. Implementation support and Educational Content	Feedback on prototypes of implementation education	Co-design clinicians	G	2	PI	Microsoft Teams	60 mins	Review and refine prototype materials
	2. Bronchiolitis Implementation Package	Bronchiolitis educational materials	Co-design clinicians	G	3	PI	Microsoft Teams	60 mins	Determine educational needs from a regional/rural perspective. Resolve conflicting feedback
	2. Bronchiolitis Implementation Package	Parent discharge information	Co-design parents	G	1	PI	Microsoft Teams	60 mins	Define key discharge information
	2. Bronchiolitis Implementation Package	Parent discharge information	Co-design parents	G	2	PI	Microsoft Teams	60 mins	Refine and finalise materials
	2. Bronchiolitis Implementation Package	Parent discharge information	Co-design parents	G	3	PI	Microsoft Teams	60 mins	Refine and finalise materials
	2. Bronchiolitis Implementation Package	Discharge information video	Co-design parents & clinicians	G	4	PI	Microsoft Teams	30 mins	Evaluate video for accuracy and relevance to regional/rural settings
	3. Online benchmarking and feedback	Development of clinical indicators for benchmarking; performance feedback reports	Co-design clinicians	G	5	PI	Microsoft Teams	60 mins	Define key benchmarking data, report features and usability needs
	3. Online benchmarking and feedback	Refinement of prototypes	Co-design clinicians	G	6	PI	Microsoft Teams	60 mins	Insight into perspectives of the prototype design, functionality and modifications needed, to guide revisions to the benchmarking report prototype
**Phase 3:** Usability testing of RART-Bronch platform	Usability testing of RART-Bronch platform	Task-based ‘walk throughs’	Co-design clinicians & additional clinicians	I	7	PI	Microsoft Teams	30 mins	Clinicians will interact with the platform using a think-aloud approach, providing real-time feedback
**Phase 5:** Evaluation of the co-design process	Evaluation of the co-design process	N/A	Co-design clinicians	I	8	PI	Microsoft Teams	60 mins	Clinician and researcher perspectives on engagement, contributions, and overall experience in shaping the RART-Bronch platform

Reflexive notes will be kept throughout the sessions. To facilitate iterative refinement, a structured feedback loop will be established throughout the co-design process. Following each co-design session, feedback from clinicians and parents will be categorised into key domains such as usability, content accuracy, and accessibility. Minor modifications will be addressed asynchronously via email, whereas substantial changes requiring further discussion will be resolved in follow-up meetings. To support transparency and ongoing engagement, a summary of how feedback has informed the development process will be shared with participants.

Given the diverse professional backgrounds of participating clinicians and the varied literacy levels of parent participants, specific strategies will be employed to tailor feedback integration. Parent-facing content will be reviewed by parents from diverse backgrounds to ensure the materials are clear, accessible, culturally appropriate, and relevant to their information needs. Clinicians will review clinical accuracy and applicability to hospitals in regional and rural settings.

### Platform development

1. **Implementation support**
  - Initial content development of implementation support

The development of implementation educational content will be led by implementation scientists and educational psychologists within the research team and will build on previously used implementation science training materials.^34^ Content will focus on hospital-specific improvements and local adaptations, particularly addressing rural and regional barriers. Educational resources such as videos, PowerPoint slides and handouts, will be finalised through the co-design process.

#### Co-design meeting 1 (clinicians): review content and format

Clinicians will provide feedback on the format, length, and usability of the proposed content, with a focus on packaging it effectively for the target audience -rural and regional-based clinicians, focused on quality improvement. Feedback will inform revisions and the development of prototype resources.

b. Feedback on prototypes of implementation education

Following the initial content review, clinician participants will be granted access to the training videos and PowerPoint slides before the second co-design meeting.

#### Co-design meeting 2 (clinicians): Review and refine prototype materials

This meeting will focus on refining the prototype materials, focusing on content relevance, presentation length, and feasibility for training clinical leaders. Clinicians will assess usability with final materials developed through iterative discussion. A follow up meeting may be held if major changes are needed.

2. **Bronchiolitis education**

The bronchiolitis education package will build upon materials developed and evaluated in the PREDICT bronchiolitis cRCT.^25^ It will feature:

- Bronchiolitis educational materials for clinicians, such as PowerPoint slides and evidence handouts.
- A discharge information handout for parents.
- A clinician-facing discharge information video.
  - **Bronchiolitis educational materials**

The educational content will be updated to align with the 2025 revision of the PREDICT Australasian bronchiolitis guideline^4^ and targeted for use in regional and rural healthcare settings.

#### Co-design meeting 3 (clinicians): evaluating the bronchiolitis implementation package

Clinicians will review the educational materials in advance and provide feedback on content structure for different clinician roles and experience levels. They will also assess suitability for regional and rural settings, and feasibility for staff training.

3. **Parent discharge information**

A discharge information sheet for parents of infants presenting to the emergency department (ED) with bronchiolitis will be developed through co-design to ensure clarity, accessibility, and inclusion of diverse perspectives. A single standardised advice sheet will be used for all families, regardless of whether the infant is admitted or discharged from the ED, to ensure consistent messaging and reduce confusion. The co-design process will involve 7-14 parents and three researchers attending an orientation session and four online meetings over three months.

#### Co-design meeting 1 (Parents): define key discharge information

A content expert will present the updated evidence, guiding parents through a typical ED /ward journey to identify key information needs. Parents will review existing discharge materials, explore barriers to delivering effective discharge information in regional and rural settings and provide feedback to inform initial prototypes for refinement in later meetings.

#### Co-design meetings 2 and 3 (parents): refine and finalise materials

Parents will review and refine discharge information prototypes, aiming for consensus on materials that are clear, accessible, and suitable for diverse communities.

- Discharge information video

A scripted role-play video for clinicians will be updated from the 2017 PREDICT bronchiolitis cRCT,^25^ to incorporate new guideline evidence and feedback from co-design groups, ensuring relevance to regional and rural settings. The draft will be reviewed asynchronously before a final 30-minute meeting.

#### Co-design meeting 4 (clinicians and parents): evaluate video for accuracy and relevance to regional/rural settings

Clinicians will assess the video’s clinical accuracy and applicability to regional and rural settings. Parents will evaluate its clarity, completeness, and relevance to their information needs.

4. Online benchmarking and feedback
  The RART-Bronch platform will support hospitals in entering de-identified patient data and receiving benchmarking reports to assess compliance with bronchiolitis guideline recommendations. Additionally, it will provide actionable, clinician-level feedback to facilitate improvement efforts and contextually targeted implementation strategies.
  - Development of clinical indicators for benchmarking
    Building on the multidisciplinary bronchiolitis guideline collaboration and previous audit and feedback work,^22^ we will develop clinical indicators to assess adherence to evidence-based practices. These indicators will cover the use of ineffective therapies and management processes, such as salbutamol, steroids, antibiotics, adrenaline, and chest x-rays, as well as the appropriate use of high flow nasal cannula therapy (HFNC therapy).
    A REDCap database, housed at [blinded for review], will store site audit data and incorporate inbuilt data quality checks to ensure completeness before submission. Once tested (see usability testing section), hospitals participating in the future cRCT will enter de-identified retrospective data into REDCap via the RART-Bronch platform.
  - Development of benchmarking performance feedback reports

Benchmarking reports will be designed using principles from human factors design, audit and feedback literature, and theory-informed approaches, with additional input from co-design participants. Audit and feedback has been found to be most effective when baseline adherence to guidelines is low, delivered repeatedly by a clinical champion in both verbal and written formats, includes clear targets and action plans^24,35,36^ and when there is facilitation to support the use of audit and feedback.^36^ An action implementation toolbox incorporated in the implementation education materials, will support the use and delivery of reports, with suggested strategies to address identified barriers.^37^

Hospitals will submit de-identified data to the central REDCap database via a secure link through the RART-Bronch platform. Data visualisation software, Power BI,^38^ will be integrated to generate feedback reports, estimating adherence as a binary variable for each clinical indicator. Each report will present hospital-specific adherence rates alongside de-identified comparisons. Where possible, reports will be generated automatically within 24 hours of data submission.

A human-centred design approach will guide development to ensure reports meet the needs and preferences of end-users.

### Co-design of benchmarking reports

Prior to finalising the benchmarking report design, a 45–60 min co-design meeting will be conducted by the researchers via Microsoft Teams with members of the Clinician Co-Design group.

#### Co-design meeting 5 (Clinicians): defining key benchmarking data, report features, and usability needs

Clinicians will provide input on the content, structure, and usability of web-based feedback reports, including preferred data presentation, design elements, and decision-making support. Feedback will inform the final report design to ensure usability and clinical relevance.

### Refinement of prototypes

Co-design findings will inform a design guide for benchmarking reports. A design team, including experts in human-centred design, audit and feedback and PowerBI,^38^ will review requirements and address design challenges. Mock-ups will be presented to the clinician co-design group in a 45–60-min meeting.

#### Co-design meeting 6 (clinicians): feedback on the prototypes design and functionality

Clinicians will provide feedback on the prototype’s design, functionality, and required modifications, guiding subsequent revisions. The qualitative data collected from these sessions will be analysed using the same strategies outlined previously.

### Prototype testing

A Power BI developer will build user-specific benchmarking report prototypes showing hospital performance on low-value care and HFNC use, with de-identified comparisons and decision-support guidance. Clinicians will test prototypes via individual task-based walkthroughs recorded on Microsoft Teams, using a think-aloud,^39^ and cognitive task analysis approach to assess usability and workflow integration.^40^

### Phase 3: usability testing of the RART-Bronch platform (months 11–12)

Following the completion of the implementation educational materials, bronchiolitis implementation package, and audit and benchmarking reports, these elements will be integrated into the RART-Bronch platform prototype. The usability testing phase will assess the platform’s design, functionality, and ease of use while identifying potential barriers to implementation in regional and rural hospital settings. Findings from this phase will inform refinements to ensure the platform is accessible, user-friendly, and fit for purpose before final deployment. To balance potential bias, usability testing will involve clinicians who participated in the co-design group, along with an additional 8–10 clinical staff from regional and rural hospitals across multiple states, who were not involved in the co-design process. This approach allows assessment of both fidelity to co-design decisions and independent evaluation of usability.

A multi-method approach will be employed to evaluate the usability of the RART-Bronch platform:

### Usability testing components

**I. Task-based ‘walk-throughs’ with think-aloud protocol** Clinicians will interact with the platform using a think-aloud approach^39^ providing real-time feedback.

**II. Quantitative usability survey** All clinicians participating in usability testing will complete a structured survey assessing the platform’s purpose, appearance, usefulness, content and impact. Responses will be analysed to identify areas for improvement prior to the final launch of the RART-Bronch platform (see Supplementary Material S1).

### Phase 4: Final refinements and study closure (months 13–23)

Usability testing findings will guide final refinements, ensuring the platform is technically and clinically validated. The research team will document changes, track decision-making, and prepare reports for stakeholders on usability and feasibility outcomes.

Key activities for this phase:

1. **Integration of feedback:** Incorporate usability and co-design feedback to improve navigation, functionality, and feasibility.
2. **Technical and content validation:** Confirm accuracy of educational content, reliability of tools (for example, the REDCap database), and overall platform performance.
3. **Stakeholder engagement:** Share evaluation findings with clinicians, hospital administrators, and funders, to align with future implementation objectives.
4. **Preparation for future implementation:** Finalise and document the platform for use in a future cRCT to improve bronchiolitis management in regional and rural hospitals.

**Data collection:** Data will be collected through multiple sources to capture refinements, validation outcomes, and dissemination efforts:

- **Feedback integration records** – Log of iterative changes based on usability and clinician feedback.
- **Technical reports** – Documentation of final validation processes.

### Phase 5: evaluation of the co-design process (months 20–23)

This phase will assess clinician engagement, perceived impact, and insights for best practice in the co-design of an online platform providing implementation support, educational materials and audit and feedback mechanisms.

**Data collection methods:**

- *Semi-structured interviews* will explore clinicians’ experiences of the co-design process with thematic analysis identifying key barriers and enablers to engagement
- *Patient Engagement in Research Scale (PEIRS) survey*, will assess transparency, communication, and influence on decision-making. Survey data will be analysed descriptively (supplementary material S2).
- *Co-Design Engagement Forms* will be completed by a researcher during each session to capture attendance, engagement, preparation, and contribution quality (supplementary material S3).

## Dissemination of findings (month 23)

Findings from this study will be shared through multiple channels to ensure broad engagement and impact. Collaboration with PREDICT and partners will support national adoption of the RART-Bronch platform.

## Data analysis

A mixed-methods approach will be used to analyse qualitative and quantitative data collected during the co-design and usability testing (Table 3).

**Table 3:** RART-Bronch data collection, analysis and storage

Stage of study	Participants (clinician or parent)	Aim	Data collection	Data recording methods	Tool employed	Data analysis
Phase 1: Establishing Co-Design Groups	Co-design clinicians	Recruit clinicians to co-design the RART platform	Consent Contact Details Demographics	Online REDCap survey	N/A	N/A
	Co-design parents	Recruit parents to co-design parent-facing content	Consent Contact Details Demographics	Online REDCap survey	N/A	N/A
Phase 2: Co-Design of the RART-Bronch platform	Co-design clinicians	Develop educational materials and audit & benchmarking tools	• Co-design meetings • Iterative feedback loops	Teams recording Transcription Note taking Asynchronous email	Co-design meeting guides Co-design engagement form	• Qualitative content analysis • Feedback integration • Reflexive analysis and decision-making tracking
	Co-design parents	Develop and refine parent discharge information	• Co-design meetings • Iterative feedback loops • Think-aloud exercises	Teams recording Transcription Note taking Asynchronous email	Co-design meeting guides	• Qualitative content analysis • Feedback integration • Reflexive analysis and decision-making tracking
Phase 3: Usability Testing of the RART-Bronch platform	Co-design and usability clinicians	Recruit usability clinicians; assess functionality, feasibility, and acceptability	• Task-based scenario ‘walk throughs’ • Usability survey	Teams recording Transcription Note taking Asynchronous email Usability survey (REDCap)	Observation data and usability survey	• Completion rates, errors, time • Qualitative content analysis • Quantitative descriptive analysis, usability problems, user satisfaction
Phase 4: Final Refinements & Study Closure	Co-design and usability clinicians	Refine platform based on feedback	• Feedback Integration Records • Technical Reports • Stakeholder Dissemination Materials	Feedback records Reports	Final feedback logs and discussion records	• Thematic Analysis of Final Feedback • Descriptive Reporting: Summary of refinements • Impact Evaluation
Phase 5: Evaluation of the Co-Design Process	Co-design clinicians	Assess clinician engagement in co-design	• Individual interviews and PEIRS survey	Transcription Note taking Asynchronous email Survey (REDCap)	Meeting guide; PEIRS survey; engagement assessment form	• Thematic analysis of interview data • Analysis of PEIRS data • Descriptive analysis of engagement data • Triangulation of findings

### Qualitative analysis

Qualitative data from co-design meetings, usability testing, and interviews will be audio-recorded, transcribed verbatim, and analysed using NVivo.^41^ A structured content analysis will identify themes related to usability, feasibility, and implementation challenges. Two researchers will code data independently, resolving discrepancies through team discussion. Reflexive notes will support analysis by documenting decision-making and consensus points.

The coding framework will be refined iteratively to ensure consistency across clinician and parent feedback. Themes will be categorised under usability (for example, platform navigation, accessibility), feasibility, and content clarity. Parent and clinician data will be analysed separately to ensure targeted refinements.

### Quantitative analysis

Survey data from usability testing, PEIRS, and co-design engagement forms will be analysed using descriptive statistics in Stata.^42^ The following metrics will be assessed:

- **Task completion and errors:** users’ ability to navigate the platform and complete predefined tasks.
- **Usability issues:** frequency, type, and severity of problems, categorised by their impact on workflow.
- **User satisfaction:** perceptions of usability, feasibility, and clinical utility.
- **User engagement:** responses to the PEIRS, reflecting involvement across seven domains.
- **Clinician co-design engagement:** attendance, preparation, and participation assessed via structured observation.

#### Triangulation

Findings will be triangulated to integrate performance metrics with user feedback, supporting a comprehensive understanding of user experience and guiding final refinements.

### Potential risks and mitigation strategies

The study poses minimal physical risk to participants, but two potential risks have been identified:

1. Emotional upset among parents: Parents may experience emotional discomfort when reflecting on their infant’s hospitalisation.
  *Mitigation:* A facilitator experienced in consumer engagement will monitor welfare during all meetings. Participants may withdraw at any time and will be offered a debrief with the study coordinator after each session.
2. Clinicians’ discomfort discussing practice variation and barriers to care: Clinicians may feel uneasy critiquing hospital processes.
  *Mitigation:* Feedback will be treated confidentially and not linked to individuals or hospitals. Anonymous feedback can also be provided via email or survey.
3. Parental discomfort in joint co-design session: Parents may feel hesitant to provide feedback on clinical practices in the presence of clinicians due to inherent power dynamics.
  *Mitigation:* Only one co-design session will involve both parents and clinicians. This session will be led by an experienced facilitator, and parents will have the option to provide feedback confidentially or outside the joint session to ensure their perspectives are fully captured.

All participants will be required to sign a confidentiality agreement as part of the informed consent process. The risk of information being shared outside the group will be discussed openly at the start of each session.

### Data management

All study data will be securely stored in accordance with institutional policies, ethical guidelines, and Good Clinical Practice (GCP) to ensure confidentiality, integrity, and security.

- **Demographic data:** Collected via REDCap, with participants assigned unique study IDs. Identifiers will be stored separately in an encrypted database.
- **Co-design meetings:** Video recordings and transcripts from Microsoft Teams will be downloaded and stored in a locked Teams Channel accessible only to the research team, then deleted from the original server.
- **Usability testing:** Observational notes and user feedback will be recorded and stored in password-protected electronic folders.
- **Survey responses:** Collected via REDCap and analysed in Stata and NVivo.
- **Co-design engagement:** Collected via Microsoft Word forms on Teams and stored securely in a locked Teams Channel.

### Data confidentiality and access

Only approved research team members will have access to study data. Identifiable information will be stored separately from research findings. All data will be retained for five years, then permanently deleted using secure data destruction software. Any hard copy files will be stored in locked cabinets and shredded at the end of the retention period.

### Data disposal

After 5 years, all electronic data, including backups, will be securely erased. Hard copy records will be destroyed to prevent unauthorised access.

## Discussion

Bronchiolitis remains the most common reason for hospitalisation among infants, particularly in regional and rural settings, where access to paediatric specialists and intensive care facilities is limited. Although evidence-based guidelines exist for bronchiolitis management, widespread practice variation remains and provision of low-value care contributes to prolonged hospital stays, increased healthcare costs, and potential patient harm.^1,12^

This study aims to co-design and evaluate the usability of the RART-Bronch platform, an online interactive tool designed to improve bronchiolitis care in regional and rural hospitals. By building on existing proven strategies and integrating implementation science principles, including audit and feedback mechanisms, the platform seeks to support clinicians in implementing best-practice guidelines while also empowering parents with accessible, evidence-based information.

The human-centred co-design approach ensures that the platform is targeted to end-user needs, addressing unique challenges in regional and rural hospitals, such as workforce constraints, resource limitations, and variable paediatric clinical experience levels. Stakeholder input will enhance the platform’s usability, feasibility, and potential for sustained adoption beyond the research setting.

Upon completion, the RART-Bronch platform will be evaluated in a future cRCT across 30 regional and rural hospitals, assessing its impact on clinical practice, patient outcomes, and knowledge translation in bronchiolitis management.

## Strengths and limitations

A key strength of this study is its evidence-based approach, which builds on successful targeted implementation strategies to translate current guideline recommendations into improved bronchiolitis management. The co-design process ensures that the RART-Bronch platform is clinically relevant, user-friendly, and targeted to the needs of regional and rural hospitals. The mixed-methods evaluation provides a comprehensive assessment, while the platform will enable scalability and offers potential for broader adoption in paediatric emergency care.

However, there are several anticipated challenges. Usability may be affected by technological barriers, such as limited digital literacy or infrastructure, in some hospitals. Additionally, recruiting a sufficiently diverse group of parents for the co-design process may prove challenging. First Nations parents will be identified through Indigenous liaison officers in regional and rural areas to guide culturally appropriate consultation and ensure inclusion of feedback from First Nations people. Despite these challenges, the structured co-design and usability testing approach will help optimise the platform for real-world implementation.

## Conclusion

This study addresses a critical gap in the implementation of evidence-based bronchiolitis management in regional and rural settings, where health inequities are exacerbated by limited access to paediatric expertise, infrastructure, and clinical support tools. The RART-Bronch platform represents a model for how co-designed, contextually targeted implementation interventions can enhance clinical decision-making and reduce low-value care in resource-constrained environments. Beyond bronchiolitis, the findings from this project will offer valuable insights for other regional and rural implementation initiatives seeking to embed evidence-based care through scalable, user-centred online platforms. By contributing new knowledge on the co-design, usability, and implementation of strategies to improve care in non-metropolitan contexts, this study has the potential to inform future improvement efforts across a broad range of acute paediatric and general healthcare conditions.

## Supplementary information

Supplementary Information

## Data Availability

Data sharing is not applicable to this article as no datasets were generated or analysed during the current study.
