# Supplementary material for: Co-design and evaluation of a regional and rural translation bronchiolitis platform: study protocol
Source: Pediatr Res. 2025 Nov 3;99(7):2451–61. doi: 10.1038/s41390-025-04503-1 (PMC13485683; doi:10.1038/s41390-025-04503-1)
Supplement: Supplementary file 1 [file 41390_2025_4503_MOESM1_ESM.pdf]

Supplemental Material S1.

Usability Evaluation Questionnaire for the RART Platform

Instructions:

Thank you for participating in this usability evaluation of the RART platform. Please answer the following questions based on your experience using the platform. If a question does not apply, select **N/A**. Your feedback will help refine the platform for future implementation.

Section 1: Background Information

1. **Professional Role:**
    - Medical Specialist
    - General Practitioner
    - Emergency Department Clinician
    - Nurse
    - Other (please specify): _______________
2. **Years of Clinical Experience:**
    - 0-5 years
    - 6-10 years
    - 11-15 years
    - 16+ years
3. **Experience with Digital Health Platforms:**
    - Never used
    - Limited use (occasional user)
    - Moderate use (use digital tools regularly)
    - Extensive use (frequent use and implementation experience)

Section 2: Platform Usability

4. **Purpose of the Platform**
    - In a few words, what do you think is the main purpose of the RART platform?

---

5. **Navigation and Ease of Use**
   Please rate your experience using the platform:

Question	Strongly Disagree	Disagree	Neutral	Agree	Strongly Agree	N/A
The platform is easy to navigate	☐	☐	☐	☐	☐	☐

Question	Strongly Disagree	Disagree	Neutral	Agree	Strongly Agree	N/A
The structure and layout are intuitive	☐	☐	☐	☐	☐	☐
Information is easy to find	☐	☐	☐	☐	☐	☐
The design is visually appealing	☐	☐	☐	☐	☐	☐
The font size and colour contrast support readability	☐	☐	☐	☐	☐	☐

6. **Task Completion and Functionality**
   Please rate the following functionalities based on your experience:

Functionality	Strongly Disagree	Disagree	Neutral	Agree	Strongly Agree	N/A
Accessing and downloading educational materials is easy	☐	☐	☐	☐	☐	☐
Entering data into the REDCap database is straightforward	☐	☐	☐	☐	☐	☐
Generating feedback reports is simple	☐	☐	☐	☐	☐	☐
The platform provides useful benchmarking data	☐	☐	☐	☐	☐	☐
The platform performs efficiently with minimal loading delays	☐	☐	☐	☐	☐	☐

Section 3: Feasibility and Acceptability

7. **Impact and Usefulness**

Question	Strongly Disagree	Disagree	Neutral	Agree	Strongly Agree	N/A
The platform supports clinical decision-making	☐	☐	☐	☐	☐	☐
The benchmarking feature is relevant to my practice	☐	☐	☐	☐	☐	☐
The platform is a useful tool for regional and rural hospitals	☐	☐	☐	☐	☐	☐
I would recommend this platform to colleagues	☐	☐	☐	☐	☐	☐
I anticipate using the platform regularly in my practice	☐	☐	☐	☐	☐	☐

8. **Potential Barriers to Implementation**
   What challenges do you foresee in implementing the RART platform in your clinical setting? (Select all that apply)
   - o Limited internet access
   - o Lack of time for training
   - o Resistance to new technology
   - o Unclear integration with existing workflows
   - o Other (please specify): _______________

9. **Suggested Improvements**
   What features or modifications would enhance the usability and effectiveness of the platform?

Section 4: Overall Evaluation

10. **Overall Satisfaction**

Question	Very Dissatisfied	Dissatisfied	Neutral	Satisfied	Very satisfied	N/A
How satisfied were you with the RART platform overall?	☐	☐	☐	☐	☐	☐

11. **Final Comments**
    Do you have any additional comments or recommendations?

Thank you for your time and valuable feedback! Your input will directly contribute to refining the RART platform for optimal usability and implementation.

Supplementary material S2.

Patient Engagement In Research Scale - PEIRS

Name: ______________________________

Date: ______________________________

Your project's name: ______________________________

____

INSTRUCTIONS: Thinking about your experience as a patient partner in the project, please respond to the statements by ticking only one box for each statement. If you are unsure about which option to choose for a statement, please give the best response you can. This questionnaire may take you about 10 to 15 minutes to complete.

Procedural Requirements

The following fourteen (14) statements are about your general experiences throughout the project.

PR1. I was interested in the issue(s) being researched in the project

Strongly Agree	Agree	Neutral	Disagree	Strongly Disagree
☐	☐	☐	☐	☐

PR2. The research team members were properly introduced to each other

Strongly Agree	Agree	Neutral	Disagree	Strongly Disagree
☐	☐	☐	☐	☐

PR3. The number of patient partners on the research project team seemed appropriate

Strongly Agree	Agree	Neutral	Disagree	Strongly Disagree
☐	☐	☐	☐	☐

PR4. I understood the objective(s) of the project

Strongly Agree	Agree	Neutral	Disagree	Strongly Disagree
☐	☐	☐	☐	☐

PR5. I <u>agreed</u> with the objective(s) of the project

Strongly Agree	Agree	Neutral	Disagree	Strongly Disagree
☐	☐	☐	☐	☐

PR6. I understood how I could contribute to the project

Strongly Agree	Agree	Neutral	Disagree	Strongly Disagree
☐	☐	☐	☐	☐

PR7. I received sufficient explanation about the project

Strongly Agree	Agree	Neutral	Disagree	Strongly Disagree
☐	☐	☐	☐	☐

PR8. I understood my ethical responsibilities for the project

Strongly Agree	Agree	Neutral	Disagree	Strongly Disagree
☐	☐	☐	☐	☐

PR9. In general, I had sufficient opportunities to contribute to the project

Strongly Agree	Agree	Neutral	Disagree	Strongly Disagree
☐	☐	☐	☐	☐

PR10. I was able to perform my tasks for the project

Strongly Agree	Agree	Neutral	Disagree	Strongly Disagree
☐	☐	☐	☐	☐

PR11. I participated in making decisions about the project

Strongly Agree	Agree	Neutral	Disagree	Strongly Disagree
☐	☐	☐	☐	☐

PR12. I received sufficient updates about the project

Strongly Agree	Agree	Neutral	Disagree	Strongly Disagree
☐	☐	☐	☐	☐

PR13. Communication within the research team was clear throughout the project

Strongly Agree	Agree	Neutral	Disagree	Strongly Disagree
☐	☐	☐	☐	☐

PR14. The project was worth the time I spent on it

Strongly Agree	Agree	Neutral	Disagree	Strongly Disagree
☐	☐	☐	☐	☐

Convenience

The following four (4) statements are about how convenient it was for you to contribute throughout the project.

CN1. I had the opportunity to provide input into selecting my tasks for the project

Strongly Agree	Agree	Neutral	Disagree	Strongly Disagree
☐	☐	☐	☐	☐

CN2. My preferences for meetings (such as time, duration, location, and format) were considered when planning meetings

Strongly Agree	Agree	Neutral	Disagree	Strongly Disagree
☐	☐	☐	☐	☐

CN3. Throughout the project, I had sufficient time to complete my tasks for the project

Strongly Agree	Agree	Neutral	Disagree	Strongly Disagree
☐	☐	☐	☐	☐

CN4. I had opportunities to express my views

Strongly Agree	Agree	Neutral	Disagree	Strongly Disagree
☐	☐	☐	☐	☐

Contributions

The following four (4) statements are about your contributions throughout the project.

CT1. I contributed by providing my perspective

Strongly Agree	Agree	Neutral	Disagree	Strongly Disagree
☐	☐	☐	☐	☐

CT2. My contributions were a good use of my time

Strongly Agree	Agree	Neutral	Disagree	Strongly Disagree
☐	☐	☐	☐	☐

CT3. I shared my knowledge within the project team

Strongly Agree	Agree	Neutral	Disagree	Strongly Disagree
☐	☐	☐	☐	☐

CT4. My workload in the project was manageable

Strongly Agree	Agree	Neutral	Disagree	Strongly Disagree
☐	☐	☐	☐	☐

Team Environment and Interaction

The following five (5) statements are about the research environment and interaction throughout the project.

T1. Throughout the project, I felt accepted as a member of the research project team

Strongly Agree	Agree	Neutral	Disagree	Strongly Disagree
☐	☐	☐	☐	☐

T2. I was an equal partner in the research project team

Strongly Agree	Agree	Neutral	Disagree	Strongly Disagree
☐	☐	☐	☐	☐

T3. My interactions within the research project team were positive

Strongly Agree	Agree	Neutral	Disagree	Strongly Disagree
☐	☐	☐	☐	☐

T4. There was mutual respect among the research project team members

Strongly Agree	Agree	Neutral	Disagree	Strongly Disagree
☐	☐	☐	☐	☐

T5. There was trust among the research project team members

Strongly Agree	Agree	Neutral	Disagree	Strongly Disagree
☐	☐	☐	☐	☐

Support

The following three (3) statements are about the support provided throughout the project.

SU1. I received sufficient support to contribute to the project (for example, orientation, readings, training workshops, webinars)

Strongly Agree	Agree	Neutral	Disagree	Strongly Disagree
☐	☐	☐	☐	☐

SU2. Any concerns I had were addressed

Strongly Agree	Agree	Neutral	Disagree	Strongly Disagree
☐	☐	☐	☐	☐

SU3. I was offered sufficient reimbursement for my out-of-pocket expenses (such as childcare, parking, and travel) related to the project activities

Strongly Agree	Agree	Neutral	Disagree	Strongly Disagree
☐	☐	☐	☐	☐

Feel Valued

The following three (3) statements are about your feeling of being a valued member of the research team.

FV1. The research project team appreciated my contributions

Strongly Agree	Agree	Neutral	Disagree	Strongly Disagree
☐	☐	☐	☐	☐

FV2. The research project team was open to receiving my views

Strongly Agree	Agree	Neutral	Disagree	Strongly Disagree
☐	☐	☐	☐	☐

FV3. I was offered sufficient recognition for my contributions (for example, payment, authorship, or gifts)

Strongly Agree	Agree	Neutral	Disagree	Strongly Disagree
☐	☐	☐	☐	☐

Benefits

The following four (4) statements are about the benefits of your involvement in the project.

BE1. I enjoyed being a part of the project

Strongly Agree	Agree	Neutral	Disagree	Strongly Disagree
☐	☐	☐	☐	☐

BE2. I made an impact on the decisions in the project

Strongly Agree	Agree	Neutral	Disagree	Strongly Disagree
☐	☐	☐	☐	☐

BE3. I saw how my contributions could benefit others

Strongly Agree	Agree	Neutral	Disagree	Strongly Disagree
☐	☐	☐	☐	☐

BE4. My involvement had positive impacts on my life

Strongly Agree	Agree	Neutral	Disagree	Strongly Disagree
☐	☐	☐	☐	☐

Supplementary material S3

RART Co-Design Engagement Form

Clinician Co-Design Sessions

Section 1: Observer & Session Details

Observer Name: _________________________

Date of Session: _________________________

Session Number: _________________________

Total Session Duration: _______ minutes

Actual Participants Attended: _______

Section 2: Attendance, Preparation & Retention

2.1 Attendance Status

Clinician ID	Attended Full Session	Arrived Late	Left Early	Previously Attended (Yes/No)

2.2 Preparation for the Session (if applicable)

Clinician ID	Reviewed Pre-Session Materials	Watched Assigned Video	Read Documents Prior to Session

Observer Notes on Preparation (e.g., impact of preparation on engagement, challenges, or barriers to preparation):

____
____
____
____
____
____

Section 3: Verbal Participation Metrics (Individual-Level)

3.1 Speaking Time & Contribution Quality

Clinician ID	Time Speaking[a]	Asked Questions	Provided Feedback	Silent	Quality of Contributions (1-5)[b]

Section 4: Non-Verbal Engagement (Individual-Level)

4.1 Visual Engagement (for online meetings – cameras, body language, attentiveness)

**Clinician ID**	**Camera On**	**Eye Contact**	**Nodding / Gestures**	**Distracted (looking away, multitasking)**	**Overall Non-Verbal Engagement (1-5)[b]**

Section 5: Engagement with Co-Design Activities

5.1 Participation in Specific Co-Design Activities

**Clinician ID**	**Reviewed Materials**	**Gave Feedback**	**Overall Engagement (1-5)[b]**

Section 6: Open-Ended Feedback

What were the key strengths of participant engagement in this session?

____
____
____
____
____
____

What challenges or barriers to engagement were observed?

____
____
____
____
____
____

What strategies could improve engagement in future co-design sessions?

____
____
____
____
____
____

[a]Co-design ordinal scale for time speaking

Rating	Score	Description

Spoke Frequently	1	Participant regularly initiated or responded to discussion, offered elaborated comments or suggestions.
Moderate Participation	2	Participant contributed at least a few times during the session with relevant comments.
Minimal Speaking	3	Participant spoke once or twice, often in response to direct prompts.
Silent	4	No verbal contribution observed.

[b]Co-Design Participation Engagement Scale

Rating	Score	Observable Participant Behaviors
Excellent	1	Highly engaged, consistently contributes valuable ideas, listens actively, and collaborates effectively. Their input directly shapes discussions and outcomes.
Good	2	Engaged and contributes meaningfully, though participation is somewhat inconsistent. Listens well and provides input but doesn't always drive discussions.
Adequate	3	Participates occasionally but with limited depth. Contributions are reactive rather than proactive, and engagement is inconsistent.
Poor	4	Minimal engagement, rarely contributes, or appears disengaged. Input is limited and has little impact on discussions.
Very Poor	5	Disengaged or resistant to participation. Does not contribute meaningfully and may hinder collaboration.
